# Should adjustment for covariates be used in prevalence estimations?

**DOI:** 10.1186/1742-5573-5-2

**Published:** 2008-01-25

**Authors:** Wenjun Li, Edward J Stanek, Elizabeth R Bertone-Johnson

**Affiliations:** 1 Division of Preventive and Behavioral Medicine, University of Massachusetts Medical School, Worcester, MA 01655, USA; 2 Department of Public Health, University of Massachusetts, Amherst, MA 01003, USA

## Abstract

**Background:**

Adjustment for covariates (also called auxiliary variables in survey sampling literature) is commonly applied in health surveys to reduce the variances of the prevalence estimators. In theory, adjusted prevalence estimators are more accurate when variance components are known. In practice, variance components needed to achieve the adjustment are unknown and their sample estimators are used instead. The uncertainty introduced by estimating variance components may overshadow the reduction in the variance of the prevalence estimators due to adjustment. We present empirical guidelines indicating when adjusted prevalence estimators should be considered, using gender adjusted and unadjusted smoking prevalence as an illustration.

**Methods:**

We compare the accuracy of adjusted and unadjusted prevalence estimators via simulation. We simulate simple random samples from hypothetical populations with the proportion of males ranging from 30% to 70%, the smoking prevalence ranging from 15% to 35%, and the ratio of male to female smoking prevalence ranging from 1 to 4. The ranges of gender proportions and smoking prevalences reflect the conditions in 1999–2003 Behavioral Risk Factors Surveillance System (BRFSS) data for Massachusetts. From each population, 10,000 samples are selected and the ratios of the variance of the adjusted prevalence estimators to the variance of the unadjusted (crude) ones are computed and plotted against the proportion of males by population prevalence, as well as by population and sample sizes. The prevalence ratio thresholds, above which adjusted prevalence estimators have smaller variances, are determined graphically.

**Results:**

In many practical settings, gender adjustment results in less accuracy. Whether or not there is better accuracy with adjustment depends on sample sizes, gender proportions and ratios between male and female prevalences. In populations with equal number of males and females and smoking prevalence of 20%, the adjusted prevalence estimators are more accurate when the ratios of male to female prevalences are above 2.4, 1.8, 1.6, 1.4 and 1.3 for sample sizes of 25, 50, 100, 150 and 200, respectively.

**Conclusion:**

Adjustment for covariates will not result in more accurate prevalence estimator when ratio of male to female prevalences is close to one, sample size is small and risk factor prevalence is low. For example, when reporting smoking prevalence based on simple random sampling, gender adjustment is recommended only when sample size is greater than 200.

## 1. Introduction

Public health studies often involve random sampling of subjects from a population defined in space and time. For example, the Behavioral Risk Factors Surveillance Systems (BRFSS) of the Centers for Disease Control and Prevention (CDC) conducts annual telephone surveys on adults living in households in the 50 states of the United States. BRFSS surveys cover many behavioral risk factors, such as cigarette smoking, sexual behaviors, and drunk driving. An important goal of such surveys is the estimation of risk factor prevalences. When sample data are analyzed, adjustments of risk factor prevalences are usually made to account for possible imbalance of covariates (also called auxiliary variables in survey sampling literature, such as gender and age) in the study samples. Post-stratification is one of the typical adjustment procedures adopted to improve the accuracy of prevalence estimators. For example, among articles recently published in BioMed Central journals, post-stratification was applied in population health surveys [1-3] and population-based case-control studies [4]. We use the example of gender adjusted estimates of smoking prevalence to motivate our study.

In theory, an estimator of smoking prevalence adjusted for known information on covariates such as the population gender proportions may be more accurate than the unadjusted (crude) one [5]. The higher accuracy occurs when the covariance between smoking and gender, which is proportional to the difference between gender-specific prevalences, is known. In practice, however, the difference between gender-specific prevalences needed for adjustment is often unknown and replaced by their sample estimates. The variability introduced by the estimates may overshadow the reduction in variance that would be achieved if the gender-specific prevalences were known, and has implications on the omnibus adoption of such procedures to obtain more accurate prevalence estimates.

To our knowledge, popular epidemiology textbooks, such as Rothman and Greenland [6], and statistical textbooks, such as Fleiss, Levin and Paik [7] do not provide guidelines with regard to when the adjustment of risk factor prevalence estimation should or should not be made. We illustrate the problem using a simple example of adjustment for gender in estimating smoking prevalence, and present an empirical guideline for when such adjustment should not be used in health survey reports.

## 2. Analysis

We begin by defining crude and gender-adjusted prevalence estimators, and reviewing the theoretical basis for covariate adjustment. We show that the adjusted estimators are a function of the difference in the gender specific prevalences. We then use Monte Carlo simulations to illustrate the impact that estimating the gender specific prevalence difference has on the accuracy of the prevalence estimators.

Suppose a survey of cigarette smoking prevalence among adult residents of a small town in Massachusetts is conducted by random digit dialing (RDD) [8] following procedures similar to those in the BRFSS [9]. For simplicity, we assume that the goal of the survey is to estimate the smoking prevalence *π *based on a simple random sample (SRS) with all adults having an equal probability of being interviewed, and cigarette smoking status (yes or no) and gender (male versus female) being reported on each sample subject.

In Massachusetts, the town list of residents, updated annually by the town government as mandated by law, provides telephone numbers and gender of adult residents. The total number of adults (*N*) and the proportion of male adult residents, *ω*, are known. We assume that a simple random sample of *n *subjects is selected from that list. We represent the smoking status for the *i*-th sample subject with an indicator random variable *Y*_*i *_(one if the subject is a smoker, and zero if a nonsmoker), and the covariate, gender, with an indicator random variable, *X*_*i *_(one if the subject is male, and zero if female). We summarize the sample and population data in Table 1.

**Table 1:** Number of subjects in a simple random sample and population by gender and smoking status

	Sample			Population		
	
	Smoking status			Smoking status		
				
Gender	*Yes*	*No*	Total	*Yes*	*No*	Total
Male	*n*_11_	*n*_10_	*n*_1•_	*N*_11_	*N*_10_	*N*_1•_
Female	*N*_21_	*n*_20_	*n*_2•_	*N*_21_	*N*_20_	*N*_2•_
	*n*_•1_	*n*_•0_	*n*	*N*_•1_	*N*_•0_	*N*

Description	Sample Notation			Population Notation		

Number of smokers	*n*_•1_			*N*_•1_		
# of male smokers	*n*_11_			*N*_11_		
# of female smokers	*n*_21_			*N*_21_		
Number of non-smokers	*n*_•0_			*N*_•0_		
# of male non-smokers	*n*_10_			*N*_10_		
# of female non-smokers	*n*_20_			*N*_20_		
Number of subjects	*n = n*_•1 _+ *n*_•0 _= *n*_1• _+ *n*_2•_			*N = N*_•1_*+ N*_•0 _= *N*_1• _+ *N*_2•_		
Proportion of male subjects	$\hat{\omega }=n_{1•}/n$			*ω *= *N*_1• _*/N*		
Smoking prevalence	$\hat{\pi }=n_{•1}/n$			*π *= *N*_•1_*/N*		
Male smoking prevalence	${\hat{\pi }}_{M}=n_{11}/n_{1•}$			*π*_*M *_= *N*_11_*/N*_1•_		
Female smoking prevalence	${\hat{\pi }}_{F}=n_{21}/n_{2•}$			*π*_*F *_= *N*_21_*/N*_2•_		

Smoking prevalence in a population is defined as the number of smokers divided by the number of members of the population, *π *= *N*_•1_/*N*. The crude (unadjusted) prevalence estimator is defined as the sample prevalence given by $\hat{\pi }=n_{•1}/n$, and can be expressed as a weighted average of gender specific prevalence estimates, as

$$\hat{\pi }=\hat{\omega }{\hat{\pi }}_{M}+\left(1-\hat{\omega }\right){\hat{\pi }}_{F}$$

where $\hat{\omega }=n_{1•}/n$ is the proportion of males in the sample, and ${\hat{\pi }}_{M}$ and ${\hat{\pi }}_{F}$ are estimates of smoking prevalence among male and female sample subjects, respectively (see Table 1). The corresponding variance is $\mathrm{var}\left(\hat{\pi }\right)=\left(1-f\right)\sigma_{y}^{2}/n$, where *f *= *n*/*N*, and $\frac{N-1}{N}\sigma_{y}^{2}=\pi \left(1-\pi \right)$ is the variance of *Y*.

A gender-adjusted prevalence estimator is obtained by replacing the sample proportion of males by the population counterpart in (1) resulting in

$${\hat{\pi }}_{2}=\omega {\hat{\pi }}_{M}+\left(1-\omega \right){\hat{\pi }}_{F},$$

and its variance may be approximated as indicated in [10]. This estimator, routinely used in public health survey reports, is the directly adjusted (or standardized) estimator. In the finite population sampling literature, it is also referred to as the poststratified estimator or poststratified estimate [11], where gender groups correspond to the post-strata.

This intuitive and straightforward procedure is widely used to reduce variance of prevalence estimates. Like the crude estimator (1), the adjusted estimator (2) is unbiased [5,10]. From (1) and (2), it may seem that only increased accuracy could result from substituting *ω *for $\hat{\omega }$. In fact, more is involved in this substitution since the crude prevalence can be computed without estimating gender specific smoking rates. This prompts the question as to whether adjusted estimators based on estimated gender specific prevalence do result in 'improved' accuracy relative to the crude estimator.

Some insight into the difference between the prevalence estimators can be gained by considering the algebraic identity, ${\hat{\pi }}_{2}=\hat{\pi }-\left(\hat{\pi }-{\hat{\pi }}_{2}\right)$. The term, $\hat{\pi }-{\hat{\pi }}_{2}$, represents the adjustment to the crude estimator. Using (1) and (2), we can express

$$\hat{\pi }-{\hat{\pi }}_{2}=\left({\hat{\pi }}_{M}-{\hat{\pi }}_{F}\right)\left(\hat{\omega }-\omega \right),$$

to show that the adjustment depends on estimates of two terms: an estimate, ${\hat{\pi }}_{D}={\hat{\pi }}_{M}-{\hat{\pi }}_{F}$ of the difference in gender specific prevalence, *π*_*D *_= *π*_*M *_- *π*_*F*_, and the difference between the sample weight, $\hat{\omega }$, and the population weight, *ω*. Variability in the estimators of gender specific prevalences may lead to extra variability in the direct adjusted estimator, offsetting the presumed gain in accuracy.

### Example: The Adjusted Estimator May Differ More from the True Prevalence than the Crude Estimator

Let us consider a simple example to illustrate how the variability in ${\hat{\pi }}_{D}$ affects the adjusted estimator. We consider a population with equal numbers of males and females, that is, *ω *= 0.5, an overall smoking prevalence of *π *= 0.2, and male and female smoking prevalences of *π*_*M *_= 0.25 and *π*_*F *_= 0.15, respectively. We further assume that the population size is sufficiently large relative to the sample size so that sampling fraction will not play an important role in the estimation. The difference in smoking prevalences in relation to male gender is thus *π*_*D *_= 0.25 - 0.15 = 0.10. We suppose that four independent simple random samples of size 100 are drawn from the population, the first two samples having 60% males and 40% females, and the last two samples having 40% males and 60% females. For each sample, we compute the crude prevalence estimator $\hat{\pi }$, the adjusted estimator ${\hat{\pi }}_{1}$ assuming *π*_*D *_is known, the adjusted estimator ${\hat{\pi }}_{2}$ using ${\hat{\pi }}_{D}$, and an additional covariance adjusted estimator ${\hat{\pi }}_{3}$ (to be discussed in the next section). The data and computed estimates are summarized in Table 2.

**Table 2:** Estimates of smoking prevalence for four possible samples from a population with 50% males, *ω *= 0.5, when the true smoking prevalence is *π *= 0.2 and male and female smoking prevalence are *π*_*M *_= 0.25 and *π*_*F *_= 0.15, respectively (*π*_*D *_= 0.10)

			Possible samples			
			
			1	2	3	4
	Prop. Male	$\hat{\omega }$	0.6	0.6	0.4	0.4

Gender-specific prevalence estimator	Male	${\hat{\pi }}_{M}$	0.2	0.3	0.15	0.3
	Female	${\hat{\pi }}_{F}$	0.25	0.1	0.2	0.1
	Difference	${\hat{\pi }}_{D}={\hat{\pi }}_{M}-{\hat{\pi }}_{F}$	-0.05	0.2	-0.05	0.2

Prevalence estimator	Crude	$\hat{\pi }$	0.2200	0.2200	0.1800	0.1800
	Adjusted (using *π*_*D*_)	${\hat{\pi }}_{1}=\hat{\pi }-\pi_{D}\left(\hat{\omega }-\omega \right)$	0.2100	0.2100	0.1900	0.1900
	Direct adjusted (using ${\hat{\pi }}_{D}$)	${\hat{\pi }}_{2}=\hat{\pi }-{\hat{\pi }}_{D}\left(\hat{\omega }-\omega \right)$	0.2250	0.2000	0.1750	0.2000
	Covariance adjusted	${\hat{\pi }}_{3}=\hat{\pi }-\frac{{\hat{\sigma }}_{x}^{2}}{\sigma_{x}^{2}}{\hat{\pi }}_{D}\left(\hat{\omega }-\omega \right)$	0.2248	0.2008	0.1752	0.1994

The gender adjusted estimator given by ${\hat{\pi }}_{1}$ (assuming *π*_*D *_is known in each sample) is closer to the true prevalence. In contrast, the direct adjusted prevalence estimator, ${\hat{\pi }}_{2}$, may be either further away from the true prevalence (Samples 1 and 3) or closer to the true prevalence (Samples 2 and 4). Over repeated sampling, the impact is an increase in the variance. One may thus naturally question whether such phenomena are simply circumstantial or have practical implications in the application of poststratification. To examine this issue, it is valuable to consider more general statistical frameworks that give rise to poststratified estimators.

### Theoretical basis for adjustment for covariates

Various approaches have been proposed in the finite population sampling literature to obtain adjusted estimates of prevalences based on simple random samples. Methods for improving estimation with known information on covariates have been discussed in model-based [12,13], model-assisted [5,10,14], calibration [15,16], or random permutation model [17,18] approaches. For the scenarios discussed here, the gender-adjusted estimator derived under the random permutation model is

$${\hat{\pi }}_{1}=\hat{\pi }-\beta \left(\hat{\omega }-\omega \right),$$

and its variance is given by

$$\mathrm{var}\left({\hat{\pi }}_{1}\right)=\left(1-\rho^{2}\right)\left[\left(1-f\right)\sigma_{y}^{2}/n\right].$$

where *f *= *n*/*N*, $\sigma_{x}^{2}$, *σ*_*xy *_is the covariance of *X *and *Y*, $\sigma_{x}^{2}$ is the variance of *X*, *ρ *= *σ*_*xy*_/*σ*_*xσy *_is the correlation coefficient (i.e., phi coefficient) [7] of smoking status (*Y*) on gender (*X*), and $\hat{\pi }$ is the crude prevalence as defined in (1). As shown in formula (4), the adjustment results in variance reduction over the simple sample proportion for any none-zero *ρ*, with the percent reduction given by (1 - *ρ*^2^) × 100%.

### Role of variance components in rate adjustment

The adjusted estimator (3) is a function of the coefficient *β*, which, in turn, is a function of variance components (*σ*_*xy *_and *σ*_*x*_) in the population. Since the gender of all adults in the population is known, $\sigma_{x}^{2}$ is also a known quantity, that is, $\sigma_{x}^{2}=\frac{N}{N-1}\omega \left(1-\omega \right)=\frac{N_{1•}N_{2•}}{N\left(N-1\right)}$. It can be shown that $\sigma_{xy}=\sigma_{x}^{2}\pi_{D}$, that is, *σ*_*xy *_is proportional to the difference between gender-specific prevalence rates so that *β *= *π*_*D*_. The resulting estimator is given by

$${\hat{\pi }}_{1}=\hat{\pi }-\pi_{D}\left(\hat{\omega }-\omega \right),$$

as in Table 2. If the difference in gender specific prevalence is known, ${\hat{\pi }}_{1}$ will always be more accurate than $\hat{\pi }$ as long as the population prevalences are not equal for males and females, i.e., *π*_*D *_≠ 0, (which implies *ρ *≠ 0 since *ρ *= *π*_*D*_/(*π*(1-*π*))).

In practice, the difference in gender specific prevalence is usually unknown, and hence *π*_*D *_is replaced by the sample estimate, ${\hat{\pi }}_{D}$. This leads to the direct adjusted prevalence, (2), which can be expressed as

$${\hat{\pi }}_{2}=\hat{\pi }-{\hat{\pi }}_{D}\left(\hat{\omega }-\omega \right),$$

as indicated in (5).

A third estimator of the prevalence is the covariance adjusted prevalence estimator given by (3) with *β *replaced by $\hat{\beta }={\hat{\sigma }}_{xy}/\sigma_{x}^{2}$ where ${\hat{\sigma }}_{xy}={\hat{\sigma }}_{x}^{2}{\hat{\pi }}_{D}$ and ${\hat{\sigma }}_{x}^{2}=\frac{n}{n-1}\hat{\omega }\left(1-\hat{\omega }\right)=\frac{n_{1•}n_{2•}}{n\left(n-1\right)}$, resulting in

$${\hat{\pi }}_{3}=\hat{\pi }-\hat{\beta }\left(\hat{\omega }-\omega \right)=\hat{\pi }-\frac{{\hat{\sigma }}_{x}^{2}}{\sigma_{x}^{2}}{\hat{\pi }}_{D}\left(\hat{\omega }-\omega \right).$$

In this case, only *σ*_*xy *_must be estimated since the known population gender distribution is used to evaluate $\sigma_{x}^{2}$. In [17], the author shows that ${\hat{\pi }}_{3}$ will have slightly smaller mean squared errors when sample sizes are relatively small (n<50). In this paper, we use ${\hat{\pi }}_{3}$ to estimate gender-adjusted prevalences and examine how the uncertainty of estimating variance components influence the accuracy of the competing prevalence estimates. A series of Monte Carlo simulations to compare the variance of the crude and the adjusted estimates was conducted for such purposes.

### Simulations

We generated a series of hypothetical populations of sizes 200, 400, 800, 1,600 and 3,200, each with proportion of males ranging from 30% to 70% (*ω *= 0.30, 0.35, 0.40, 0.45, 0.50, 0.55, 0.60, 0.65, 0.70), and with hypothetical prevalence of cigarette smoking ranging from 15% to 35% (*π *= 0.15, 0.20, 0.25, 0.30 and 0.35). The ratio of male to female smoking prevalence ranges from 1.0 to 4.0 (ratio = 1.0, 1.1, 1.2, 1.3, 1.4, 1.5, 1.6, 1.7, 1.8, 2.0, 2.2, 2.4, 3.0, 3.5, 4.0).

We evaluated the accuracy of adjusted and unadjusted prevalence estimators by comparing the average variance using over 10,000 independent simple random samples for each scenario. The sample sizes ranged from 25 to 200 (*n *= 25, 50, 100, 150, 200), corresponding to sampling fractions ranging from 1.5% to 75%. In total, 15,120 scenarios were evaluated.

**Figure 1:**
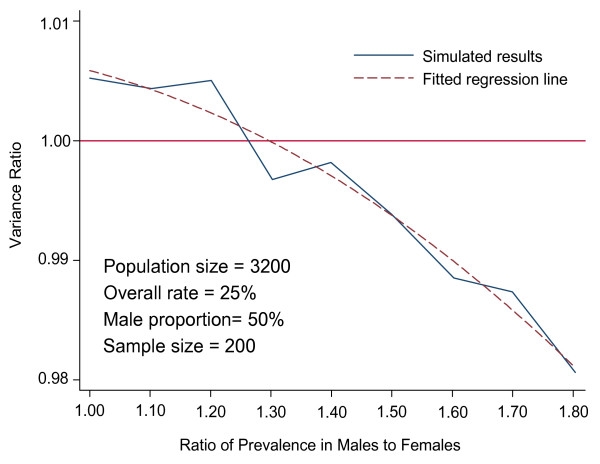
Variance ratio of adjusted prevalence to unadjusted prevalence.

To compare the variance of the prevalence estimators, we computed ratios of the variance of the adjusted estimator to the variance of the crude estimator and plotted them against the percentage of males in population, by population smoking prevalence, population size and sample size. Figure 1 contains such plots with equal gender proportions, true prevalence rate of 25%, sample size of 200 and population size of 3200. Thresholds of ratios of male to female prevalences, above which adjusted prevalence estimators have smaller expected variances, were determined graphically. Regions above the plotted lines indicate that increased accuracy will result from adjustment.

## 3. Results

**Figure 2:**
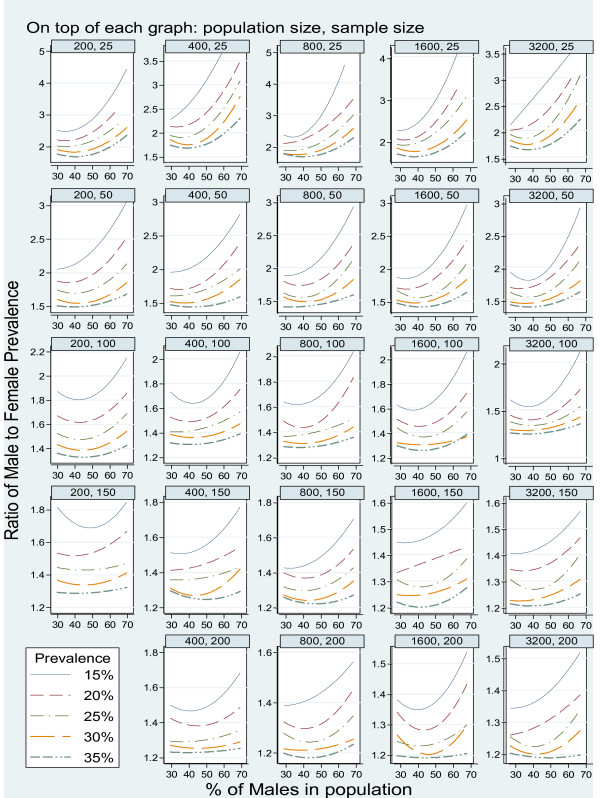
Ratio threshold for variance reduction due to gender adjustment by prevalence, population and sample sizes.

We present results graphically in terms of a set of threshold ratios of male to female prevalence. When the ratio of male to female prevalence exceeds the threshold, the adjusted prevalence estimator is more accurate than the unadjusted estimator. The estimated threshold ratios are presented in Figure 2. From the upper left plot in Figure 2, for example, the ratio threshold is approximately 2.6 when the population size is 200, sample size is 25, the overall smoking prevalence is 15%, and the population consists of equal numbers of men and women.

The variance reductions due to gender-adjustment depend on sample sizes, gender proportions, gender-specific prevalence ratios, and overall prevalence in the population. In populations with balanced gender proportions and overall prevalence of 35%, the adjusted prevalence estimators have smaller variances when gender-specific prevalence ratios are above 1.6, 1.5, 1.4, 1.3 and 1.2 for sample sizes of 25, 50, 100, 150 and 200, respectively. In populations with unbalanced gender proportions, the ratio thresholds are higher.

When the population prevalences are lower, for example, at 15% in gender balanced populations, the ratio thresholds are much higher, i.e., 2.6, 2.1, 1.8. 1.6 and 1.5 for sample sizes of 25, 50, 100, 150 and 200, respectively.

The ratio thresholds are much higher in populations with high male proportions, in particular, when sample sizes are under 150. For example, in a population of size 1,600 with 70% males and prevalence of 15%, the ratio thresholds are above 4, 3, 2.3, 1.6, and 1.5 for sample sizes of 25, 50, 100, 150 and 200, respectively.

Sampling fractions influence the variance reduction as well, with higher sampling fractions related to slightly higher threshold ratios. For example, with a sample of size 150 from a population with balanced gender proportion and prevalence of 15%, the ratio thresholds are 1.7, 1.55, 1.45 and 1.4 for populations of sizes 200, 400, 800 and 1,600 (corresponding to sampling fractions of 75%, 38%, 19%, and 9%). According to these data, in a study of a small town with 1600 adults, based on a sample of size 200, if the ratio of male to female prevalences matches the Massachusetts population ratio (1.13), gender-adjusted estimators should not be used.

A simulation program in Stata 9.2 available on our web site[19] can be used to evaluate whether an covariates should be controlled in other settings.

## 4. Discussion

Information on covariates can be used for reducing the variance of prevalence estimators across sub-populations. Our simulation results illustrate that adjustment for covariates, while designed to make estimators more accurate, may actually have the opposite effect. Although the simulations are limited to gender adjustment on estimators of smoking prevalence, the results have broad implications for other settings in epidemiological applications. The basic idea is that adjustment for covariates involves estimating regression coefficients of the outcome variable on covariates based on sample data. In theory, adjusted prevalence estimators are more accurate than crude estimators when the regression coefficient *β *or the relevant variance components (*σ*_*xy *_and $\sigma_{x}^{2}$) are known [5]. If the relationship between the covariates and the response variable is weak, the added variability due to estimating the coefficients, however, can overshadow the gain. In our example, the use of empirical estimators based on the sample covariance between smoking status and gender (${\hat{\sigma }}_{xy}$) results in a reduction in the variance of the adjusted estimator only when the ratio of male to female prevalences is sufficiently large, or when the association between smoking status and the covariate (i.e., gender) is sufficiently strong. Given the same sample size, the implication is that confidence intervals for the prevalence estimator will be wider and have poor coverage when adjustment for covariates is used. In fact, when the ratio of the male and female prevalences is lower than the thresholds indicated in Figure 2, gender-adjusted prevalence estimators are less accurate than the unadjusted ones.

Poststratification is usually viewed as a procedure to reduce confounding and minimize variance. In practice, the domain sizes (the numbers of males and females) in a sample are often mistakenly perceived as fixed numbers. Poststratification is prompted to account for "potential confounding" due to differences between sample and population gender proportions. However, such justification for poststratification contradicts the fact that the numbers of males and females vary between samples. In fact, both the unadjusted and post-stratified estimators are unbiased based on repeated sampling[10]

One of the strengths of our study is that we simulated a large number of scenarios that are similar to common situations in health surveys, where sample sizes are small and prevalences are relatively low. The ranges of the parameters used for the simulations mimic those in data from an ongoing national survey (BRFSS). According to data from 2005 BRFSS data of Massachusetts [20], the prevalence of current cigarette smoking is around 18%; the ratio of the male to female prevalence is 1.13, the ratio of adults with low annual per capita income (< $15,000) to those with high annual per ca income (>$50,000) is 2.1; the ratio of individuals with less than high school education to college graduates is 2.6; and the ratio of those aged 18 to 25 to those aged 65 or older is 3.1. The ranges of gender proportions and smoking prevalence are similar to those in 1999–2005 BRFSS data for Massachusetts. Although the sampling fractions in BRFSS surveys are generally small (<2.5%) and thus may not be important, we included scenarios with small populations and high sampling fractions to reflect those situations with over-sampling of subpopulations or geographic areas where sampling fractions may play an important role in estimation.

Based on the simulation results, we provided empirical guidelines to identify situations where adjusted prevalence estimators should not be used in place of crude ones. In certain situations, a much stronger relationship is needed for the adjustment for covariate unbalance to be warranted. For example, with a sample size of 100 and prevalence rate of 20%, a reduction in the variance of the adjusted prevalence estimator will occur only if the ratio of male to female prevalences is greater than 1.7 in a population with balanced gender proportions, or over 2.5 in a population with 70% males and 30% females. Based on BRFSS data for Massachusetts, one can conclude that post-stratification by gender may result in less accurate estimates of local smoking levels with sample size less than 200.

In this paper, we illustrated very simple scenarios where there is only one binary covariate (gender). More complicated, yet common scenarios, such as those with multiple subgroups (e.g., race-ethnicity), or multiple categorical covariates (e.g., gender, race-ethnicity and age groups), or with mixture of categorical covariates (gender) and continuous covariates (age), assuming the relationship of the covariate with the prevalence is linear, should be further investigated. In particular, the impact of variance component estimation on the variances of adjusted prevalence estimators should be evaluated. We are currently developing simulation studies that address these settings. As discussed by Kish and Anderson[21] and Särndal and Lundström [22], we expect that the post-stratification adjustment would perform better when more relevant covariates with relatively few categories for each of the post-stratifying variables are included as opposed to many categories for each of a small number of post-stratifying variables.

Our findings may have important implications on the reporting methods of public health survey data, such as the BRFSS surveys; in particular, they may impact reporting of the municipal (county, town or city)-level statistics. For example, among the 351 communities (towns or cities) of the Commonwealth of Massachusetts (2003 BRFSS survey data), 49 have a sample size ≥ 30, and only 7 have a sample size ≥ 100. If the smoking prevalence of the 49 municipals with sample sizes between 30 and 100 are to be reported based on direct estimates from the sample, our empirical guidelines suggest that gender-adjusted prevalence estimators should not be used in place of crude estimates. Instead, when sample size is greater than 100, the prevalence estimates should be adjusted for one or a few of the covariates that are related to large differences in smoking prevalence between strata, such as age group (18–44, 45–65 vs. >65), race-ethnicity (non-Hispanic black, Hispanic, other race vs. non-Hispanic white), marital status (widowed, separated, never married, divorces vs. married living together), employment status (unemployment vs. employed or not in labor force), and annual household income in thousands (<15, 15–24, 25–49, vs. ≥ 50). Modest and strong correlations between smoking and these covariates have been documented in literature. In addition, the correlations are in the range considered in this study.

Adjustment for covariates is typically not the only issue facing analyst of survey data. Other important issues in the BRFSS include nonresponses and probability weighting. Practical issues in random digit dialing surveys may result in responses not exactly matching those expected in a simple random sample from the population [6,8]. For this reason, weights that account for both sampling and response probabilities are usually applied. While these problems are practically important, they do not devalue the findings presented in this paper.

In summary, we recommend that health survey analysts not carry out adjustment on prevalence estimators without reviewing the relationship between the response and the covariates. Adjusted rates should not be used in all settings, and in particular, not when prevalence, prevalence ratios and sample sizes are small. In such settings, adjustment will lead to less accuracy of the prevalence estimates and to an illusion of statistical control. We anticipate that this problem will be aggravated by inclusion of three or more covariates that are modestly correlated with the outcome, but this problem warrants further investigation.

## Competing interests

The author(s) declare that they have no competing interests.

## Authors' contributions

WL and ES conceived the study. WL carried out the analysis and drafted the manuscript. ES and EBJ participated in manuscript preparations. All authors read and approved the final manuscripts.
